# Dataset of identified scholars mentioned in acknowledgement statements

**DOI:** 10.1038/s41597-022-01585-y

**Published:** 2022-08-01

**Authors:** Keigo Kusumegi, Yukie Sano

**Affiliations:** 1 University of Tsukuba, Graduate School of Science and Technology, Ibaraki, 305-8573 Japan; 2 University of Tsukuba, Faculty of Engineering, Information and Systems, Ibaraki, 305-8573 Japan

**Keywords:** Complex networks, Databases

## Abstract

Acknowledgements represent scholars’ relationships as part of the research contribution. While co-authors and citations are often provided as a well-formatted bibliometric database, acknowledged individuals are difficult to identify because they appear as part of the statements in the paper. We identify acknowledged scholars who appeared in papers published in open-access journals by referring to the co-author and citation relationships stored in the Microsoft Academic Graph (MAG). Therefore, the constructed dataset is compatible with MAG, which accelerates and expands the acknowledgements as a data source of scholarly relationships similar to collaboration and citation analysis. Moreover, the implemented code is publicly available; thus, it can be applied in other studies.

## Abstract (summary)

**Table:** 

Measurement(s)	acknowledgements section • acknowledged scholar
Technology Type(s)	natural language processing • social network analysis

## Background & Summary

Research is shifting to teamwork^1–3^, and collaboration is becoming more common^2^. Clarification of scholars’ contributions becomes a necessary process, which provides recognition for scholars working in large teams, supporting transparency of research output simultaneously^4^. In terms of research contributions, acknowledgements are the official statements of a scholar’s contribution, similar to co-authorship and citations^5,6^, although they are not always included in papers. As the number of co-authors increases, the number of papers including acknowledgement increases, and acknowledgement is a constitutive element of research activities^7,8^.

In this study, we focus only on acknowledged scholars. Generally, acknowledgements can include the names of people who have contributed to the research. For example, acknowledgements can include those who handled logistics in fieldwork and even family members who provided mental support. However, acknowledged individuals who are not scholars do not appear in bibliographic data and are difficult to identify. Therefore, we focus only on acknowledged scholars, although they are interesting subjects for future research.

The quantitative analysis of acknowledgements has been conducted using various types of datasets. Acknowledgement data are frequently collected manually^7–12^. Cronin *et al*. collected acknowledgement data through a questionnaire survey and argued that few formal rules exist in the acknowledgement section. Additionally, they discovered that peer interaction or communication could help lay bare the rules and dynamics of collaboration^9^. In other studies, acknowledgements were gathered from *Psychological Review*, *Mind*^8^, and *The Journal of the American Chemical Society*^7^.

In 2008, the Web of Science (WoS) began collecting acknowledgement data when funding information was included in papers^2,13,14^. WoS provides a grant number, funding organization, and acknowledgement text^15^. These large sorted data have advanced the research of funding impact^13,15–19^ to interpersonal relationships revealed from the acknowledgement statement^2,6,20,21^. However, the collection methods limiting the papers with funding might result in data bias when it comes to analyzing interpersonal relationships based on the acknowledgement statement. Paul-Hus *et al*. demonstrated that the tendency of acknowledgements varied across disciplines using WoS and mentioned that the effect of papers noting acknowledgements without funding could not be considered^2^.

As another approach of collecting acknowledgement data, automated acknowledgement extraction has been implemented^2,11,22–24^. Giles *et al*. succeeded in extracting acknowledged entities using regular expressions and support vector machines, whereas Stanford Named Entity Recognizer tools (https://nlp.stanford.edu/software/CRF-NER.html) have been applied in other works^2,23–25^. Khabsa *et al*. proposed an architecture called AckSeer, a search engine for the automatic extraction of acknowledgement statements and acknowledged entities^23^. After the extraction, they discussed name-disambiguation problems for the organization but not personal names. The lack of affiliation and scholar ID of acknowledged individuals is thought to be the reason for this, making it more challenging to identify acknowledged scholars.

This study provides a unique dataset that enables the identification of acknowledged scholars and facilitates the use of acknowledgements as part of the interpersonal information between scholars. We build a dataset of scholars acknowledged in open-access journals. After extracting the acknowledgement statement from the papers, the names of the individuals were tagged and retrieved using annotation software. These individual names go through a Microsoft Academic Graph (MAG) to be matched as identified scholars in the context of collaboration and citation. A manual review was performed on randomly selected 400 samples to validate the dataset, which exhibited a high predictive performance.

The method and curated data offer a further expansion of acknowledgement data and potential usage. The proposed method for identifying acknowledged scholars has generalizability because it requires only the acknowledged scholar’s name and paper ID for each paper, and it could be applied to other journals as long as it can be parsed in XML format. Furthermore, the proposed data serve as a critical data source to extend research in collaboration with acknowledged scholars.

The dataset of scholars mentioned in the acknowledgements is unique from the view of science of science^3,26^. Under the current practice that the notion of acknowledgements differs among disciplines and authors, the large curated dataset can be used to expand studies of collaborative activities^27^ and a new evaluation scheme of research contribution^4^. Furthermore, since the implemented code is publically available, the dataset can be expanded using the same method for other journals, as long as it is accessible to acknowledgment and paper’s information. This means that the information of acknowledged scholars, with or without funding information, can be collected in various fields.

## Methods

**Fig. 1:**
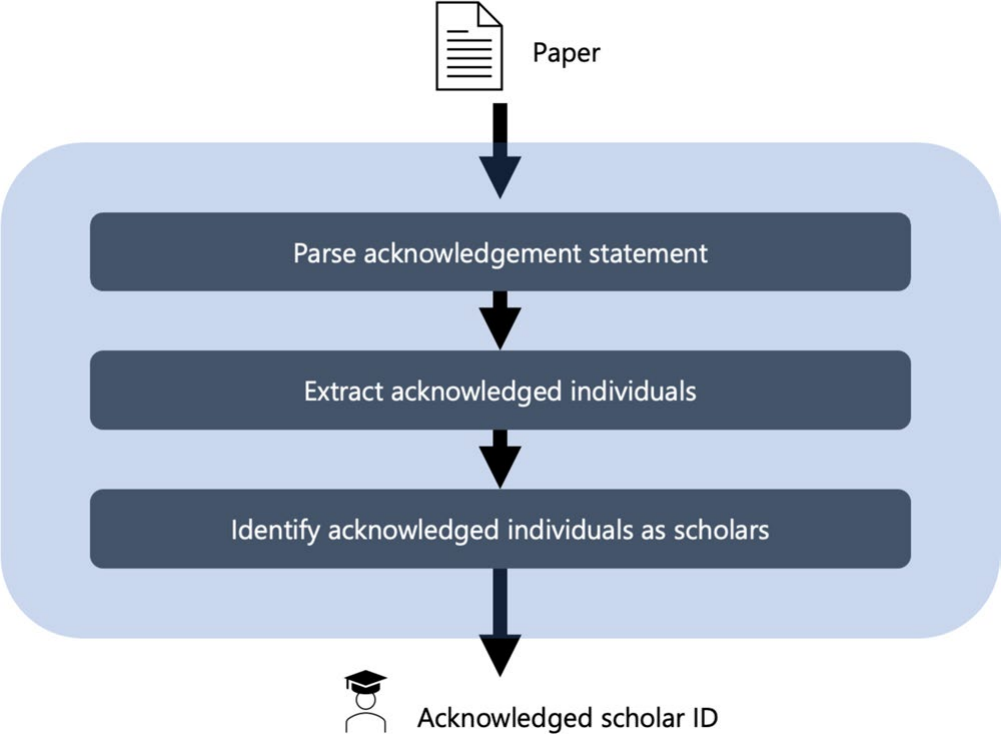
Overview of the processes from raw data to identify acknowledged scholars. With the input of text of papers, three major steps exist until the identification of the acknowledged scholars.

We constructed a dataset of scholars mentioned in the acknowledgement section of the eight open-access journals. First, we extracted the acknowledged individuals mentioned in the acknowledgement statement and matched them with the MAG to identify acknowledged scholars. Figure 1 shows an overview of the procedure. In the following subsection, we explain the algorithm used to identify acknowledged scholars.

### Parse acknowledgement statements

Acknowledgement statements were extracted from eight open-access journals: PLOS Biology (Biol), PLOS Computational Biology (Comput Biol), PLOS Genetics (Genet), PLOS Medicine (Med), PLOS Pathogens (Pathog), PLOS Neglected Tropical Diseases (NTDs), PLOS ONE, and Scientific Reports (Sci Rep). These journals were selected because of their accessibility to papers. They publish papers under the Creative Commons Attribution (CC BY) license: https://journals.plos.org/plosone/s/licenses-and-copyright for PLOS series and https://www.nature.com/srep/journal-policies/editorial-policies#license-agreement for Sci Rep. These data are available on the PLOS and Sci Rep websites in the extensible markup language (XML) format^28,29^. Similar to Larivière *et al*.^30^, we automatically downloaded the XML format of each paper and extracted acknowledgement statements using XML syntax rules. Notably, we only focused on the statements mentioned in the acknowledgement section to systematically extract information, which suggests that acknowledgement statements provided as a footnote on the title page are out of scope. Although some machine learning based approaches for extraction have been proposed^22,23^, they will remain an application of this method in future work. Moreover, these journals have been established for more than ten years, and we can assume that numerous scholars have contributed to them.

We collected 428,189 papers published between 2003 and 2021. In 2017, we crawled PLOS series paper data published between 2003 and 2016. In 2021, we crawled those published between 2017 and 2021 and Sci Rep between 2011 and 2021. We performed this based on the website specifications. For example, for the PLOS series, we accessed the website once every 30 seconds. Comput Biol was the exception, which was collected only in 2017 because the information to find papers did not exist in their sitemap (http://sitemaps.plos.cloud/journals/sitemap-index.xml) in 2021. After the parsing procedure, we found 329,480 papers, that is, 76.9% of the papers contained acknowledgements.

### Extraction of acknowledged individuals

Once we obtained the acknowledgement statement, we extracted acknowledged individuals from the text. We applied Stanford CoreNLP^31^, which is a Natural language Processing (NLP) software, to derive linguistic annotations for text, such as tokens, sentence boundaries, parts of speech, and named entities. We used this software to extract the nouns tagged with the “person” as the acknowledged individuals, similar to An *et al*.^32^ Although the names of the paper authors might appear in the acknowledgement texts, their names are removed in the latter process where the acknowledged scholars are identified via citation and collaborative relationships. Consequently, 203,428 papers had “person” in their acknowledgements, and 847,086 entities were retrieved. At this point, the individuals mentioned in the acknowledgements are not necessarily scholars.

### Identification of acknowledged scholars in MAG

After extracting acknowledged individuals, we identified the scholars among them. We used “AuthorId” in the MAG to specify scholars. MAG is big academic data containing scientific papers with citation relationships between them. It contains various types of information, such as authors, institutions, journals, conferences, and research fields. In this study, we used a snapshot of the MAG released on November 23, 2021, to extract collaborative and citation relationships. It contained 269,451,039 papers published in 49,063 journals. Notably, the MAG update was completed by the end of 2021. We believe that other service providers, such as OpenAlex (https://openalex.org/), could be used instead of MAG for future applications^33,34^.

MAG was used because of the well-constructed name-disambiguation algorithm that optimizes the accuracy of a profile by combining rich context information (example, affiliation, co-authors, year, and venue of the publication). For example, while MAG succeeded in distinguishing the two different people with identical names belonging to the same institution^35^, an author’s name represented in several patterns such as “Evan J Collins,” “E J Collins,” and “E Collins” are treated as one author with a unique AuthorID in MAG. This suggests that if these profiles can be appropriately combined, it may be possible to distinguish individual profiles with high precision and recall in our dataset. Li *et al*. built a dataset of publication records for Nobel laureates merging with MAG to realize high accuracy, dealing with the name-disambiguation problem simultaneously^36^.

Here, we identify acknowledged scholars based on the idea that acknowledged individuals may have other relationships with an acknowledging author, specifically in terms of collaborative and citation relationships. For research contribution, the criteria to be mentioned in acknowledgement vary based on academic disciplines and journal guidelines^2,30,37^. Given this blurring of the difference between acknowledgements and collaboration, a scholar receiving the acknowledgements may collaborate on other papers with the author who provided the acknowledgements. Moreover, reference lists indicate disciplines and reveal scholarly relationships via citation^38^. Citation and collaboration tend to be geographically biased, and their distributions are spatially related^39,40^, which suggests that the scholarly relationships via citation somewhat reflect the physical distance between scholars. Therefore, the acknowledged scholar’s paper was cited in the paper.

**Fig. 2:**
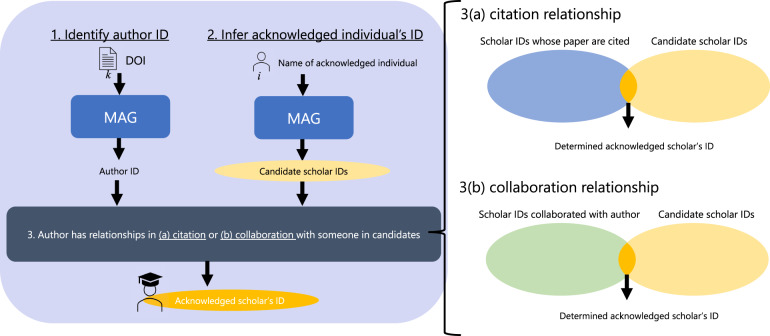
Proposed method for identifying authors and acknowledged scholars.

With these ideas, we attempt to recognize whether acknowledged scholars have collaborative or citation relationships with acknowledging authors to identify who the acknowledged is. The detailed steps (Fig. 2) are as follows.

1. **Identify acknowledging authors in MAG**. Because the collected papers have unique digital object identifiers (DOIs), we used the DOIs to extract paper information from the MAG. Therefore, the author IDs of the acknowledging papers in the MAG can be obtained through the DOIs.
2. **List the scholar IDs of the candidate acknowledged**. We obtained the acknowledged scholar IDs by exact string matching of the name with the MAG. Here, we only have the names parsed from the acknowledgement texts, and we face the name-disambiguation problem. For example, there is a case in which acknowledged individuals are not registered in the MAG, and even if the name is matched in the MAG, multiple AuthorId are suggested in several cases. Therefore, we obtained the possible scholar IDs by matching names as much as possible in this step. Simultaneously, some cases identified the names of institutions or foundations tagged by CoreNLP as “person” (example, Marie Curie and Salud Carlos III). Here, we manually verified the top 20 names frequently mentioned in the acknowledgements and removed those that appeared as names of institutions or foundations (Table 5).
3. **Narrow down acknowledged scholar IDs from the candidates**. We sought the relationships between authors and acknowledged scholars via AuthorID in MAG, assuming that authors have relationships in collaboration or citation with acknowledged scholars.
  - **Collaborative relationships**. We verified whether a candidate-acknowledged scholar ID *i* in paper *k* is the one of the collaborators of paper *k*’s authors. If *i* is the collaborator of paper *k*’s authors, we assume that this collaborator might correspond to the scholar acknowledged in paper *k*. The timing of collaboration covers all co-authorship relationships in the data, regardless of when an acknowledgement interaction occurs. This enabled us to consider both situations (that is, acknowledgement before and after collaboration). We applied this method when only one possible scholar could be identified in the procedure. We ignored the case in which more than two IDs of possible acknowledged scholars are discovered in the list of collaborators.
  - **Citation relationships**. We verified whether a candidate-acknowledged scholar ID *i* in paper *k* is one of the authors of the papers cited by *k*. If *i* exists in the list of cited authors, we assume that this author might correspond to the acknowledged scholar in *k*. Similar to the collaborative relationship, we only considered the case in which only one possible scholar could be identified.
4. **Merge** **and identify the acknowledged scholar ID in MAG**. Finally, by concatenating these two results from the collaborative and citation approaches, we determined the acknowledged scholar’s unique ID. We merged the results obtained in the (a) collaboration and (b) citation approaches. Subsequently, if the same ID is identified in both approaches, we use it as the final acknowledged scholar ID. If only the ID is identified in either of the two approaches, we use it as the final acknowledged scholar ID. If a different ID is identified in both approaches, we use neither ID and remove it from the dataset. The number of removed cases was less than 1% of the identified IDs, that is, 1,875 cases.

## Data Records

### Data structure

We built an acknowledged scholars’ dataset at the Data Bank repository at the University of Tsukuba (https://commons.sk.tsukuba.ac.jp/data_en) and Zenodo^41^. It contained eight comma-separated value (CSV) files. Each file name corresponded to a journal; the details are listed in Table 1.

**Table 1:** List of datasets for acknowledged scholars. All datasets are CSV format files containing acknowledged scholars’ IDs.

File	Lines	Short description
compbiology.csv	3,186	Acknowledged scholars in paper published by PLOS Computational Biology
biology.csv	5,189	Acknowledged scholars in paper published by PLOS Biology
medicine.csv	4,263	Acknowledged scholars in paper published by PLOS Medicine
genetics.csv	11,357	Acknowledged scholars in paper published by PLOS Genetics
ntds.csv	6,139	Acknowledged scholars in paper published by PLOS Neglected Tropical Diseases
pathogenes.csv	9,278	Acknowledged scholars in paper published by PLOS Pathogens
plosone.csv	155,461	Acknowledged scholars in paper published by PLOS ONE
srep.csv	40,693	Acknowledged scholars in paper published by Scientific Reports

All files followed the same format and included information on DOI, PaperID, AcknowledgedID, and the detected approach (Table 2). DOI and PaperID were used to identify papers, and PaperID was matched to PaperID in MAG. AcknowledgedID is the acknowledged scholar ID, and it is consistent with AuthorID in MAG. The detected approach introduced in the previous section was described by the Boolean value, True or False. If a scholar is detected by the collaborative relationship, the value of “CollaborationApproach” will be True, and the value of “CitationApproach” will be filled in the same manner. Thus, if a scholar is detected by both approaches, both values are filled as True.

**Table 2:** Data type for the acknowledged scholars.

Index	Type	Short description
DOI	String	DOI of a acknowledging paper
PaperId	Integer	PaperId of a acknowledging paper in MAG
AcknowledgedId	Integer	Acknowledged scholar’s ‘ AuthorId in MAG
CollaborationApproach	Boolean	True if a scholar is detected by collaboration relationships, otherwise False
CitationApproach	Boolean	True if a scholar is detected by citation relationships, otherwise False

Table 3 presents the descriptive statistics of the data. Generally, we collected 428,189 papers, and after the identification procedures, 235,566 scholars were matched with unique 180,375 IDs of MAG IDs. PLOS ONE is the largest dataset in which 127,551 acknowledged scholar IDs were detected from 73,869 papers. Additionally, we discovered that the average number of acknowledged scholars identified per paper was approximately two, except for Med. The majority of acknowledged scholars were detected only by collaborative relationships in all journals. The acknowledged scholars identified by both relationships followed, and the scholars identified only by citation relationships were the least case (Table 4).

**Table 3:** Descriptive statistics of each dataset. Journal names are abbreviated. “PLOS” has been omitted in the PLOS series except for PLOS ONE.

	Comput Biol	Biol	Med	Genet	NTDs	Pathog	PLOS ONE	Sci Rep
Number of identified acknowledged scholars	2,905	4,802	3,984	9,241	5,050	7,606	127,551	37,185
Number of papers including identified acknowledged scholars	1,539	2,045	1,044	4,343	2,857	4,034	73,869	20,612
Average number of the identified acknowledged per paper	2.07	2.54	4.08	2.62	2.15	2.30	2.10	1.97

**Table 4:** Numbers of detected scholar IDs by collaboration and citation approaches. Journal names are abbreviated as in Table 2.

	Comput Biol	Biol	Med	Genet	NTDs	Pathog	PLOS ONE	Sci Rep	Total
Collaboration	1433	2436	2369	4478	3084	4042	80679	19567	118088
Citation	431	826	278	1736	419	1172	15240	7161	27263
Both	1157	1706	1424	3726	1836	2936	39916	11580	64281

**Table 5:** List of names that have been manually removed because they were used as institutions or foundations.

List of institutions
Instituto de Salud Carlos III
Albert Einstein
La Jolla
Fundação de Amparo
Marie Curie
Generalitat Valenciana
Alice Wallenberg
Fundação de Amparo
Generalitat de Catalunya
Marie Skłodowska-Curie
Miguel Servet
Salud Carlos III
Sara Borrell
Severo Ochoa
KU Leuven
Susan G. Komen
Deutsche Forschungsgemeinschaft
della ricerca
Fondazione Umberto Veronesi
Ricerca Corrente
Liwen Bianji
Institut Curie
Irene Feroce
chapel Hill

## Technical Validation

### Predictive performance

We validated the dataset using two steps: the predictive performance of extracted individual names from the acknowledgement statement with CoreNLP and the reliability of identified scholars via MAG.

For name extraction, we validated how accurately CoreNLP can extract all scholars’ names from acknowledgement statements (recall) and how accurately extracted scholars were a person (precision). The sample size for this evaluation was 400, with a 5% margin of error^42^. We manually verified the personal names in the acknowledgement section; the recall and precision were 0.936 and 0.977, respectively. Ann Arbor (place) is a typical example of false-positive name used for calculating precision. Some names are false negatives that failed to be parsed by CoreNLP, which calculates recall.

After identifying acknowledged scholars, we manually verified the reliability of the identified scholars using MAG. The acknowledged scholars identified via MAG have affiliations. We verified whether the affiliation obtained via MAG was consistent with the affiliation information mentioned in the original paper to ensure that the predicted acknowledged scholars were the same person as the original scholars mentioned in the acknowledgement section. We selected 423 samples, which is the satisfied sample size of validation^42^, randomly selected from assigned acknowledged scholars. Although 400 is the minimum sample size determined by the same method as that of the aforementioned sample size^42^, an additional 23 samples were collected to compensate for the unknown result described in the following. Subsequently, eight independent master’s or doctor’s course students at the University of Tsukuba manually verified them, surveying their names and profiles on the Internet.

One hundred and nineteen of the 423 samples had explicit information about affiliation in the acknowledgement of the original papers. For the 281 samples without explicit affiliation in the acknowledgement statement, we verified whether one of the affiliations of the identified acknowledged scholars was the same as one of the authors of the original paper. If the affiliation is listed in the original acknowledgement statement along with the name, we assume that the affiliation is listed in the scholar profile in MAG. Moreover, the search results of acknowledged names on the Internet were also taken to ensure precision.

After synthesizing the above information, we discovered that 0.985 of 400 samples were accurately estimated, and only six cases were incorrect. Notably, 23 samples remained unknown owing to poor information on the Internet; therefore, we could not use them for validation. We could not calculate the recall of identified scholars because of the false-negative cases. To detect false-negative cases, information about “who should have been acknowledged” is needed that is hard to recognize.

### Network analysis

Besides data performance, the primary network analysis has been performed over the dataset. Analyzing interpersonal acknowledgements as a network helps to understand the structure of the interpersonal acknowledgement relationships similar to collaboration and citation networks^5,6^. We briefly show the property of the acknowledgement network with the curated data.

We built a directed acknowledgement network where each node represents either author or acknowledged scholar, and each edge represents acknowledgements from an author to a scholar. It should be noted that a node may be both an author and an acknowledged scholar. We placed a directed edge from individual *i* to individual *j* if there is a paper authored by *i* acknowledges *j* in the acknowledgement section. The elements of the adjacency matrix are given by

1 $${a}_{ij}=\left\{\begin{array}{ll}1 & {\rm{if}}\;i\;{\rm{acknowledges}}\;j,\\ 0 & {\rm{otherwise}}.\end{array}\right.$$

**Fig. 3:**
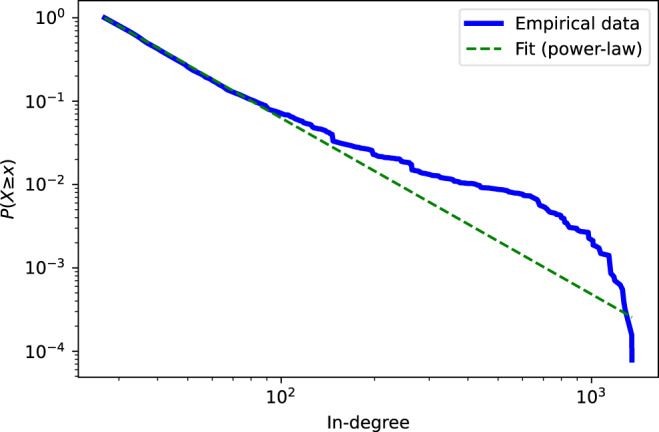
Complementary cumulative distribution of in-degree for the acknowledgement network. The parameter *α* of the power-law distribution is estimated as 3.11.

The in-degree of node *i* is ${K}_{i}^{{\rm{in}}}={\sum }_{j=1}{a}_{ji}$. Figure 3 shows the complementary cumulative distribution of the in-degree ${K}_{i}^{{\rm{in}}}$ for the acknowledgement network. We see that the empirical acknowledgement network is indeed fat-tailed. This implies that few individuals have received a huge number of acknowledgements from the authors, while most scholars have been acknowledged only a few times. Similar results have been reported in the previous studies that the distribution follows a power-law^22,24^.

As for the comparison, a statistical test^43^ indicates that power-law *P*(*x*) ~ *x*^−*α*^ with *α* = 3.11 provides a significantly better model of the data than exponential distribution when we fit the entire distribution (*p* = 0.001). However, log-normal distribution $P(x) \sim \frac{1}{\sqrt{2\pi }\sigma x}\exp \left[-\left({\left({\rm{ln}}x-\mu \right)}^{2}/2{\sigma }^{2}\right)\right]$ with *μ* = −686.3 and *σ* = 18.1 is not significantly better fit than the power-law (*p* = 0.37).

Table 6 shows the ten highest in-degree scholars. The in-degree is not equal to the acknowledged count directly because the number of authors per paper is not considered. For example, considering that one is acknowledged once in a paper of a hundred authors and the multiple single-author papers frequently acknowledge the other, the in-degree of the former would be higher than that of the latter. Table 7 shows the ten most highly acknowledged scholars counted per paper. Taking the three highly acknowledged scholars counted per paper (e.g., Takaji Wakita, Shizuo Akira, and Bert Vogelstein) as an example, we checked their acknowledged context. While some mentioned meaningful discussions, such as “We thank Bert Vogelstein and Kenneth Kinzler for very helpful discussions and breast cancer DNA samples.” (10.1371/journal.pmed.0050114), most of them are described as data providers (Takaji Wakita and Bert Vogelstein) and a supplier of mice (Shizuo Akira). To mitigate the effect of the number of authors, another network design, such as taking the weight of edges by the number of authors, should be considered in future works concerning interpersonal acknowledgement networks.

**Table 6:** The ten highest in-degree scholars.

Name	AcknowledgedId	In-degree
Heather Thorne	1979004069	1350
Eveline Niedermayr	2010482067	1348
Judi Maskiell	2054520798	1284
Maggie Angelakos	2616126658	1284
Teresa Selander	2305142036	1271
Helena KemilÃ¤inen	2615861737	1264
Michael Stagner	2577585844	1259
Pei Chao	2790030099	1237
Ursula Eilber	294419845	1188
Irja Erkkilä	2614792144	1181

**Table 7:** The ten most highly acknowledged scholars counted per paper.

Name	AcknowledgedId	Acknowledged count
Takaji Wakita	1974321678	73
Shizuo Akira	2149472920	51
Bert Vogelstein	679456835	46
Feng Zhang	2256777311	42
Noboru Mizushima	1985327407	41
Charles M. Rice	2235486152	40
Roger Mundry	345639720	39
Bernard Moss	2104435105	34
Norbert Perrimon	174839232	32
Kamil Ugurbil	1996768038	31

Furthermore, we computed the clustering coefficient (CC) of the network. CC measures the completeness of the neighbourhood of a node in a network; the higher the CC value at a node, the higher the probability that its neighbours will be connected together^44^. The average CC for the network is 0.053. It is pretty low and indicates that neighbouring nodes do not tend to cluster.

## Usage Notes

A major feature of this dataset is that the identified acknowledged scholar IDs are compatible with MAG, the widely used academic graph dataset in scholarly citation and collaboration-network analysis. Therefore, this adaptable acknowledgement dataset is expected to accelerate the application of bibliometric analyses to acknowledgements.

The curated datasets offer a new perspective on scientific research, specifically (1) expansion of research on academic activities and (2) development of scholar-evaluation schemes.

First, several studies have maintained that personal acknowledgement data can help reveal scholarly social relationships^14,20,25,45^. For example, Laudel demonstrated that one-third of the collaboration is rewarded by acknowledgement, and approximately half of the collaboration is mentioned in neither the co-author list nor acknowledgement section^10^. In this case, considering acknowledged scholars and the author as contributors simultaneously offers a more realistic quantitative analysis of academia in the era of collaboration^2^. As a specific application of the curated data from this perspective, a new type of field-of-study relationship or diversity could be studied, considering the acknowledged contributions of scholars.

Second, the identified acknowledged scholar datasets may help develop scholar-evaluation schemes. The evaluation of scholars’ research contributions is ethically, socially, and technically challenging. In addition to authorship, diverse information, such as contribution roles and acknowledgement debt^46,47^, is expected to estimate scholars’ contributions. As part of the evaluation system for authorship, contributor roles taxonomy (CRediT), which clarifies the role of authors in the process of submitting papers, has been proposed^4,48^ and adopted for more than 20 publishers, such as PLOS and Springer. This represents the transparency and responsibility of authors in scientific papers^4,48,49^. Similarly, we believe that the recognition of acknowledged scholars will provide precise research contribution^50^ and enrich academic contribution, which will assist in visualizing the recognition of a scholar working with a large team^4^.

This study has limitations. We succeeded in collecting a considerable amount of data from multiple open-access journals because of NLP tools. However, the predictive performance of extracting acknowledged names is not perfect, even when we manually removed the names of institutions or foundations. Additionally, possible biases exist in the collected data depending on whether the source data comes from open-access journals. In the process of detecting acknowledged scholars, we extracted only those scholars who were either collaborators or had citation relationships. Therefore, scholars who did not have these relationships, such as proofreaders and scholars providing datasets/code/expertise on specific methods without registration in MAG may have been overlooked.

These problems could be addressed by combining other datasets or another approach to identifying a person with machine learning^51,52^ for further enhancement of this data. Methods that address the name-disambiguation problem focus on authors, and these should be modified and extended to apply to scholar names that appear in acknowledgement. We believe this study, which focuses on establishing new prospective data of acknowledgement provided that citation and collaborative relationship results in identifying acknowledged scholars, serves as helpful reference and base data because such acknowledgement data at the level of scholars are valuable.

Finally, although eight specific open-access journals were used as source data, the method used for identifying acknowledged scholars has generalizability with the implemented code for this algorithm^53^. The described method can be applied to any other article as long as we can obtain this information because the core function of this method requires only an acknowledgement statement and DOI to identify scholars in MAG. For other types of articles or journals, the acknowledgement section could be automatically extracted by noting the different structures of XML. While we focus on the statement only described in the acknowledgement statement, the application of another approach using machine learning^22,23^ might enable us to discover acknowledgement described in other sections, such as the footnote or introduction. Once retrieved, the statement goes through CoreNLP to extract the entities and the following proposed algorithm of identification with the code.

## Data Availability

To identify individuals from acknowledgement statements, the NLP software CoreNLP^31^ was used. Script files were created using Python programming with version 3.9, to build a dataset that is available in Zenodo^53^.
